# Malaria risk factors amongst forest going populations in Mondulkiri Province and Kampong Speu Province, Cambodia: a large cross-sectional survey

**DOI:** 10.21203/rs.3.rs-5291817/v1

**Published:** 2024-11-01

**Authors:** Ingrid Chen, Dyna Doum, David J McIver, Vanney Keo, Pisey Vong, Sophak Pech, Vanny Meth, Sour Bun, Kimheng Pen, Sopagna Chea, Kanha Ly, Kry Hok, Siv Sovannaroth, Jafit Ting, Diana Lovin, Joanne Cunningham, Elodie Vajda, Allison Tatarsky, Neil F Lobo

**Affiliations:** Malaria Elimination Initiative, Institute for Global Health Sciences, University of California, San Francisco; Health Forefront Organization; Malaria Elimination Initiative, Institute for Global Health Sciences, University of California, San Francisco; Health Forefront Organization; Health Forefront Organization; Health Forefront Organization; Health Forefront Organization; Mondulkiri Provincial Health Department; Mondulkiri Provincial Health Department; Kampong Speu Provincial Health Department; Kampong Speu Provincial Health Department; Kampong Speu Provincial Health Department; Cambodia National Malaria Center; Malaria Elimination Initiative, Institute for Global Health Sciences, University of California, San Francisco; University of Notre Dame; University of Notre Dame; Malaria Elimination Initiative, Institute for Global Health Sciences, University of California, San Francisco; Malaria Elimination Initiative, Institute for Global Health Sciences, University of California, San Francisco; University of Notre Dame

**Keywords:** malaria, malaria elimination, vulnerable population, forest malaria, forest dweller, vector control, mosquito, volatile pyrethroid, spatial repellent, insecticide-treated clothing

## Abstract

**Background:**

Cambodia strives to eliminate all species of human malaria by 2025, requiring that foci among forest-exposed populations in remote settings be addressed. This study explores malaria risk factors amongst forest-exposed groups in Mondulkiri and Kampong Speu Provinces, Cambodia as part of a multi-stage study on novel bite prevention tools (Project BITE).

**Methods:**

A serial cross-sectional survey explored the demographics, housing structure openness, mosquito bite prevention habits, and gaps in protection amongst three target groups: forest goers who work in the forest, forest dwellers who live in the forest, and forest rangers who patrol forested regions. Malaria prevalence data was collected at three time points using rapid diagnostic tests (RDTs) for febrile individuals and qPCR for all participants. Infection locations and travel patterns of P. falciparum-infected individuals were analyzed for clustering and the potential movement of infections.

**Results:**

2,935 participants were enrolled between October 2022 and February 2023, consisting of 1,093 (37%) forest goers and 1,787 (61%) forest dwellers across both provinces, and 55 (5%) forest rangers in Mondulkiri province. Most worked outdoors as farmers, day laborers, and forest collectors, and reported going to the forest five to seven days a week. For housing, 29% and 39% of participants reported living in partially open primary and secondary structures, respectively. The main methods of mosquito bite protection used were insecticide-treated nets, wearing long sleeves, and burning mosquito coils, with gaps in protection during the daytime and outside at night. All febrile individuals had negative RDT test results. For qPCR, 24 *P. falciparum* infections (<1%) were detected among forest goers and dwellers, clustered in Pu Trom and Pu Nhav villages in Mondulkiri Province, and Banteay Roka and Banteay Roka Kirisenchey (M) villages in Kampong Speu Province. *P. vivax* cases were detected (216 cases, 5%) across all enrolled villages. Only two infections were found in forest rangers.

**Conclusion:**

Malaria elimination strategies for forest-exposed populations in Cambodia should focus on vector intervention strategies that offer protection during the day and outside at night, and the use of drug-based strategies to clear subpatent infections, targeting forest goers and dwellers in villages where cases are detected.

## Background

The Greater Mekong Subregion has made tremendous progress towards its goal to eliminate human malaria by 2030 [1]. This region has the highest prevalence of *Plasmodium falciparum* parasites that are resistant to artemisinin-class antimalarial drugs, and intensive efforts to eliminate this species of malaria regionally have contributed to a 97% reduction in malaria deaths, and 77% reduction in all malaria cases between 2012 and 2022 [2]. Within this region, Cambodia, the epicenter of drug-resistant *P. falciparum* parasites, is undergoing last mile efforts for elimination, with only 1,384 infections detected in 2023 [3] and no malaria-related deaths reported in country since 2017. Remaining infections are concentrated in remote locations in forested areas, often along international borders [4]. To meet its goals to eliminate human malaria nationally by 2025 [5, 6], Cambodia will need to clear malaria infections among high-risk populations in highly forested locations where access to healthcare is poor [7–9] and outdoor malaria transmission is common [10–13].

This project is a part of a multi-stage study, Project Bite Interruption Towards Elimination (BITE), on novel mosquito bite prevention tools in a “forest pack” intended to guide the last mile efforts in Cambodia and potentially other locations challenged by outdoor malaria transmission. In earlier phases of the study a pilot study was conducted in Mondulkiri province investigating malaria risk factors [14], the efficacy of insecticide treated clothing and spatial repellents to prevent mosquito bites [15, 16], and their acceptability among users [14]. Results were applied to this project, which is a large-scale risk factor assessment to characterize high-risk populations in Mondulkiri and Kampong Speu provinces. Villages included in this study are some of the highest incidence malaria settings remaining in Cambodia [12], where malaria elimination efforts have been challenged by a high prevalence of outdoor-biting vectors including *An. dirus, An. minimus,* and *An. maculatus mosquitoes* [7, 8, 11, 12, 17]. In addition to risk factor investigation this study includes malaria diagnosis data using rapid diagnostic tests (RDTs) and qPCR, providing insight on the nature of infections (symptomatic versus asymptomatic), whether they are geographically clustered, and whether human movement is a factor affecting transmission patterns. Additional outcomes from the parent study, including the rollout and user acceptability of mosquito bite prevention tools to this cohort, will be reported elsewhere.

## Methods

### Study design

This was a serial cross-sectional survey among forest-exposed individuals. Data was collected at three timepoints: October 2022 (Timepoint 0; T0) for baseline data collection, and two follow-up timepoints in December 2022 (T1), and February 2023 (T2). Due to loss to follow-up, additional participants were recruited in T1. Most risk factors were identified from the baseline survey for new participants recruited at T0, such as their demographics, housing structures, previous history of malaria infection, and malaria prevention tools in use prior to study initiation. New participants recruited in T1 had basic demographic data collected, and for some variables we used results collected in T1 and T2, for example time spent in the forest, to gain further insight on whether those risk factors changed throughout the malaria season. At all three timepoints, malaria diagnosis was also conducted using RDTs for febrile individuals and dried blood spots collected for all individuals for subsequent identification of parasite infection using qPCR [18]. All positive cases identified were mapped to villages where participants resided, to check for clustering patterns. For *P. falciparum,* travel patterns were investigated for potential associations between human movement and parasite incidence. This was not done for *P. vivax* because incident infections could either be new ones or relapses.

### Study location and population

**Figure 1:**
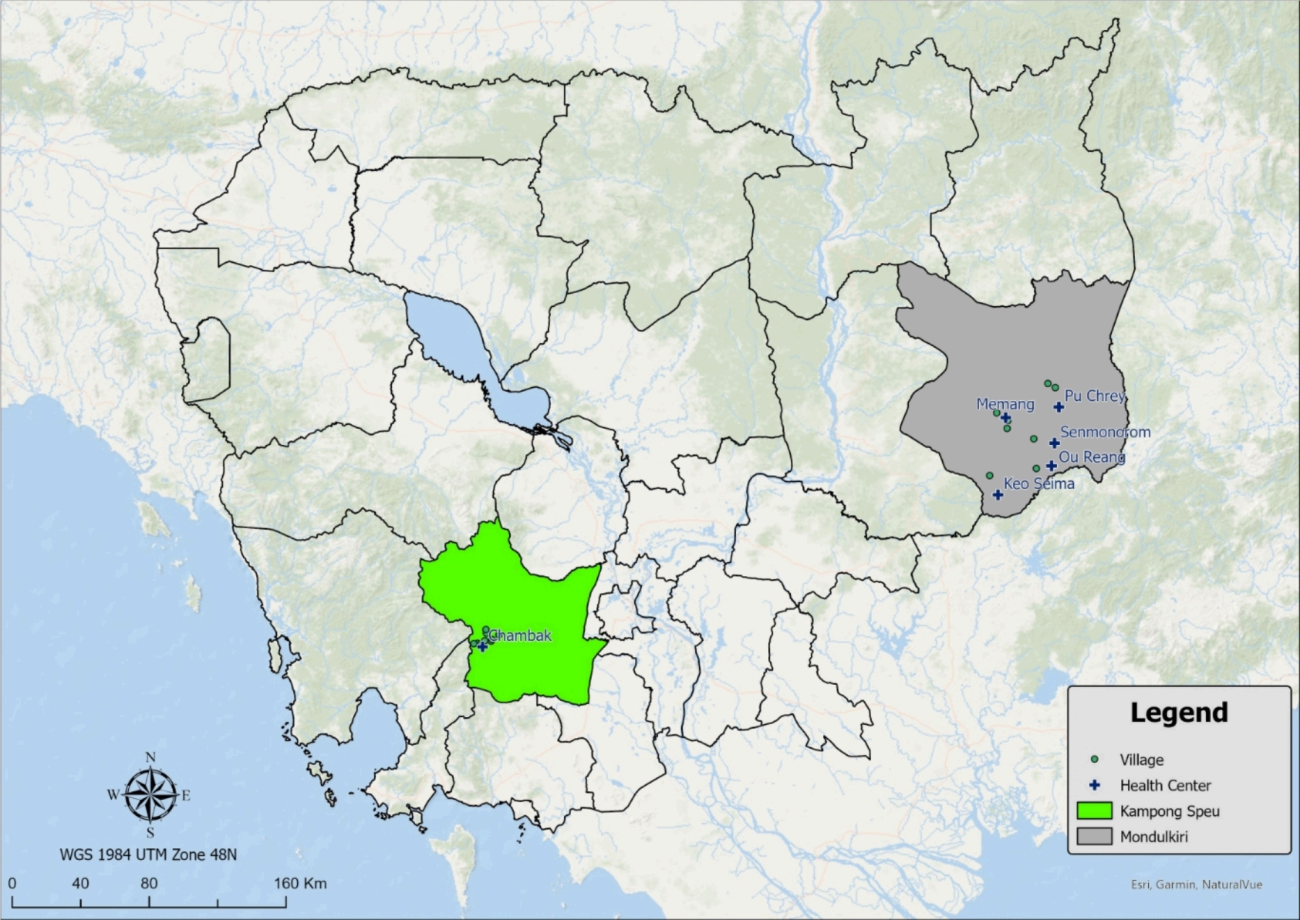
Map of Cambodia with study provinces, villages, and health center locations

This study took place in Mondulkiri Province and Kampong Speu Province, Cambodia (Fig. 1), where malaria peak case rates occur during the rainy season from August to January. The targeted study population included three high-risk populations for malaria: forest goers, forest dwellers, and forest rangers [10, 13–17], the first two of which were defined during the pilot phase of this study [14]. Forest goers were individuals who lived at least 1 kilometer from the forest, traveling to the forest regularly for seasonal farming, hunting, or foraging (mushrooms, vegetables, and resin) or seasonal migration for gem mining, logging, and plantation work [11, 14]. Forest dwellers survived on subsistence farming, living in the forest or within 1 kilometer of its fringes in a traditional house in a village for at least part of the year. Many forest dwellers also had a more open, temporary structure in the farm or forest that they migrated to during planting and harvesting seasons referred to as secondary living structures [14]. Forest rangers were recruited from Mondulkiri province only; they worked for government or wildlife and conservation agencies that protect the forest and areas near international borders, staying in ranger stations or outdoor hammocks up to 16 nights per month when they were on patrol [14].

Villages for the study were selected based on consultation with the Cambodian National Center for Parasitology, Entomology and Malaria Control (CNM), who identified locations with actively identified *P. falciparum* foci that would likely benefit from receiving vector control forest packs for the parent study. “Village” refers to geographic regions that contain residential living structures or ranger stations in forested or non-forested locations, which were defined based on an administrative level within the Cambodian governmental system.

### Inclusion Criteria

Individuals eligible for inclusion had to:

1. Reside in a village selected for the parent study, which required that they:
  1. Were accessible by automobile or motorbike from September through January
  2. Had access to routine malaria data
  3. Had leaders with existing relationships with both the government and implementing partner
2. Meet the definition of one of the three target populations at the time of enrollment:
  1. Forest goer: lived outside the forest (more than 1 km from forest edge) with self-reported travel into the forest at least 15% of the time (approximately 1 day a week)
  2. Forest dweller: lived in a village in the forest (or within 1 km of forest edge)
  3. Forest ranger: actively conducted forest patrol activities and was based at a ranger station
3. Be willing to meet study staff on a monthly basis for study follow-up activities, including meetings and the collection of finger prick blood samples for malaria testing.
4. Be age ≥ 3 years
5. Provide informed consent if aged ≥ 18 years, or obtain consent from guardian if participant is < 18 years of age
6. Speak Khmer and/or Bunong language

### Recruitment

Individuals from the three target populations were recruited using different methods. For forest goers and dwellers, the study team worked closely with the local government, implementing partners, village chiefs, and other influential members of the communities, to gain support for the study and determine the best methods for participant recruitment. To recruit forest rangers, our study team partnered with the Wildlife Conservation Society (WCS), engaging with manager-level forest rangers in three ranger stations in Mondulkiri province only, because there were very few ranger stations operating in Kampong Speu.

When recruiting prospective participants, the study team introduced themselves and checked if individuals met the study inclusion criteria. Those eligible and interested to partake in the study were provided with detailed information about the study including the purpose of the project, potential risks and benefits of participation, project duration, and expectations of participants for the study. The study team emphasized that individuals could refuse enrolment in the study or remove themselves from the study at any time without repercussion. After these details were provided, informed consent was sought in Khmer or Bunong. Those able to read were given the informed consent form, and those unable to read had the form read aloud to them. Those who provided informed consent were then enrolled in the study. Eligible individuals from each village were recruited until the target number of participants was reached.

### Data collection and analysis

At T0, a survey questionnaire was administered to all participants capturing village, household, and individual-level demographic information, housing construction, and use of vector control tools at baseline. At T1 and T2, follow-up surveys were conducted that included basic demographic data, self-reported travel, and time spent in the forest over the past two weeks. Surveys are in the supplemental information; S1 was for village demographics at all timepoints, S2 was for detailed household and individual demographic information at T0, and S3 was for simpler information at T1. All survey instruments were developed in English, translated to Khmer, and field tested. Written Bunong language was recently developed and very few people can read or write the language, so when conducting surveys in this language the data collector verbally translated each question and recorded answers in Khmer.

Surveys were conducted by staff of the Cambodia-based NGO Health Forefront Organization (HFO), a primary research partner for this program. Data were collected using Open Data Kit (ODK) with smartphones to limit data entry errors, and were uploaded to a cloud server daily. When data collection was complete, the database was downloaded onto password secured computers and analyzed by a member of the study team using Stata (StatCorp Version 14).

Data were analyzed descriptively, with results stratified by target populations as these may have different risk profiles for malaria. Data was also analyzed by province, and where differences were found results were separated accordingly. For risk factors that were not expected to vary with time, such as demographic data and housing structure construction, data from all participants in T0 and all new participants in T1 were combined to reflect the backgrounds of all enrolled individuals. For time spent in the forest, data from T1 and T2 were analyzed separately as malaria incidence was expected to decrease throughout the study period.

When each survey was conducted, RDTs for malaria were administered to participants who said they had a fever (temperature above 37 °C) during the survey. Dried blood spots were collected from all participants for later diagnosis using qPCR, which entailed extracting and amplifying DNA using a sensitive method that can detect infections below the detection limit of RDTs and microscopy [18]. Positive qPCR-detected infections were speciated, and the characteristics of infected individuals and their village of residence were analyzed, as well as self-reported travel patterns for those infected with *P. falciparum* malaria.

### Sample size calculation

The final sample size implemented was based on requirements for the parent study looking at malaria prevalence at each timepoint following the distribution of various vector control tools and the number of the number of forest packs available and distributed. Overall at least 2,100 participants were included at each time point (total of 2935).

## Results

An overview of villages, households, and individuals enrolled during the study is shown in Table 1. Participant demographics are then described, followed by several risk factors for malaria including housing characteristics, time spent in the forest, and mosquito bite prevention tools used. Next, we summarize individual malaria history, cases identified using RDTs and qPCR, the locations of positive identified cases, and travel patterns for incident *P. falciparum* infections.

### Village demographics

The villages enrolled for forest dwellers and goers are described below. A total of 18 villages were enrolled, with nine in Mondulkiri and nine in Kampong Speu province (Table 2). Villages in Mondulkiri had higher average numbers of households (199) and individuals (865) as compared to Kampong Speu, which had an average of 107 households and 375 individuals per village. For accessibility by car, this was lower in Mondulkiri, at 56%, as compared to 100% in Kampong Speu. Per inclusion criteria, all villages were accessible by motorcycle during the rainy season when the study took place. The number of Khrom, smaller clusters of households which are often separated from the main village but still included as part the village, was close to three per village for both provinces. Village data was not applicable to forest rangers, who were recruited from three ranger stations in Mondulkiri province.

### Housing demographics

Forest goers and dwellers enrolled at T0 were asked about their household amenities, and findings were similar between the two risk groups and provinces. For primary water source the most common answer as bottled water used in approximately 30% of households, almost half (47%) had flush toilets while approximately one third (34%) had no toilet, and more than half (61%) of households had electricity (table S1).

### Participant Demographics

The study enrolled 2,935 individuals, including 1,093 (37%) forest goers, 1,787 (61%) forest dwellers, and 55 (2%) forest rangers (Table 3). Demographics were similar when comparing provinces, except that Mondulkiri Province had 59% of individuals of the Bunong ethnic group and 1% of other ethnicities while in Kampong Speu, all participants were Khmer (Table S2). The predominant ethnic group overall was Khmer, although approximately a third of forest goers and dwellers were Bunong, with a small number of participants in each group being from other minority ethnic groups. The majority of participants were ages 26–45, with an average age of 33. Forest goers and rangers were predominantly male (59% and 96%, respectively), whereas dwellers reflected a smaller proportion of males compared to females (41% males). For position in their household, the vast majority of rangers were head of household, as well as more than half of the forest goers enrolled. For those who were not head of household, approximately half of participants were adults who were the son or daughter of the head of household, and results were similar for each target group.

Participants enrolled at T0 were given a list of options on their sources of income. The main income sources reported by participants required spending time outside in the forest, with the most common income source being a farmer, which was represented by almost half of all participants (47%), including 26% of forest rangers who sometimes had more than one job (Table 4). For forest dwellers and rangers, other common sources of income included day labourers, which could include work in unskilled construction (e.g., rubber industry, rice mills), and forest collectors or foragers who gathered supplies from the forest.

### Household amenities and structure openness

Participants in all risk groups enrolled at T0 were asked about how open their living structures were, to determine their vulnerability to mosquito bites when spending time indoors. Primary living structures were similar across provinces (table S4) and across risk groups, with the majority of structures (71%) being closed with walls and a ceiling or roof (Table 5). The next most common answer (28%) were partially open structures with two to three walls and a ceiling. When asked whether participants had a secondary structure in the forest or farm, this was higher (57%) in Mondulkiri province as compared to Kampong Speu (20%) (table S3), and different between risk groups with most (95%) of forest rangers having a secondary structure, as compared to half (52%) of forest goers and 31% of forest dwellers. Most of these only had a ceiling (44%), posing risks of getting mosquito bites, with the next most common structure being enclosed (33%). Structure characteristics were similar when comparing risk groups.

### Time spent in the forest

To understand the risk factors for getting malaria in the forest, participants at T0 were asked how often they go to the forest during the dry and rainy seasons. Results were similar in both provinces, with an average of approximately six days per week. This was approximately seven days a week for forest dwellers in both dry and rainy seasons (as they most often lived directly inside the forest), five to six days per week for forest goers with slightly higher frequency during the rainy season, and approximately five days a week for forest rangers.

During follow-up surveys at T1 and T2, participants were asked how many days they spent in the forest in the past week (Table 6). Results were similar between provinces (table S5) and timepoints, with 85% of participants reporting going to the forest during the past week, with higher frequencies seen for forest rangers (98%) compared to forest dwellers (93%) and forest goers (71%). Those who went to the forest spent an average of 5 days in the forest every week, with 88% of forest dwellers reporting that they went to the forest daily, as compared to 59% of forest rangers and 34% of forest goers.

### Baseline mosquito bite prevention tools used

At T0, participants were asked about the mosquito bite prevention tools they used, not including the tools that were provided as part of the parent study after this survey. At a household level for forest dwellers and goers, almost all (97%) owned a bednet, most of which were treated with insecticides (79%) (Table 7). More than half (66%) of households enrolled also owned at least one hammock net of which most (84%) were treated with insecticides. Bednet and hammock ownership were very similar when comparing forest goers and dwellers.

Participants at T0 were also asked about which mosquito bite prevention tools they used indoors or outdoors, during the day and night. Almost all participants reported using protective measures inside at night. When outside at night, protection was often used, especially for rangers (91%) as compared to forest dwellers (74%) and goers (66%). During the daytime, less protection from mosquito bites was used, with similar levels seen indoors and outdoors amongst all risk groups. Forest rangers had more than 80% protection outdoors, while dwellers had closer to 70% and goers around 55%.

**Figure 2:**
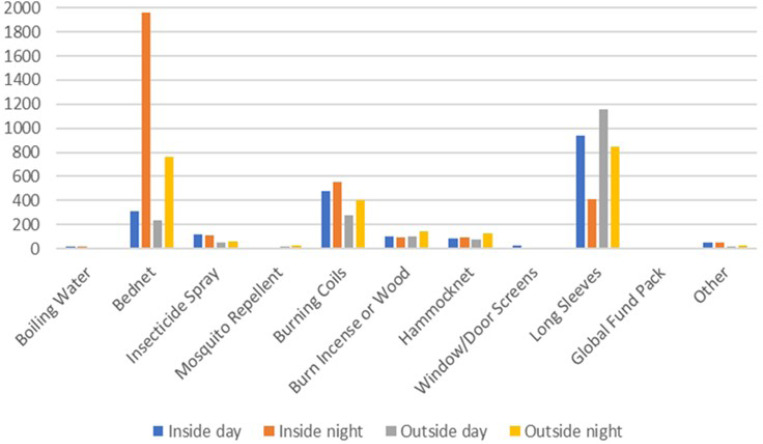
Mosquito prevention methods used in villages

**Figure 3:**
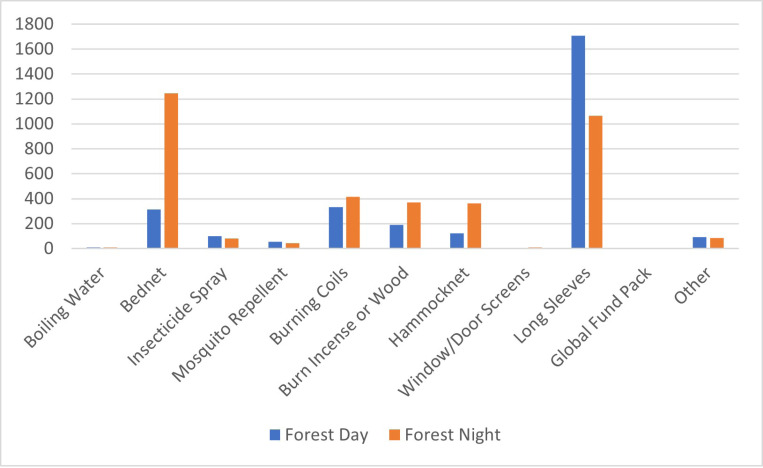
Mosquito prevention methods used in the forest

When asked about specific tools used at different times and locations, results were similar when comparing their use in villages (Fig. 2) and in the forest (Fig. 3) across all target groups. Sleeping under insecticide-treated nets was the most common method of protection, both indoors and outdoors at night, while wearing long sleeves in all circumstances except for being inside at night, when bednets were presumably preferred. The third most common method reported was burning coils. A Global Fund pack comprised of an insecticide-treated hammock net and topical repellent distributed by health workers and funded by the Global Fund to fight AIDS, Tuberculosis and Malaria.

### Travel patterns

To understand general levels of mobility and travel, participants at T0 were asked how far they travel to buy necessities. Most participants reported having purchasing activities within the range of 500m from their primary residency location, especially forest dwellers. Forest rangers generally reported having a greater range of travel distance, with 45% of them reporting buying things from places located more than 5 km from their residency location (Table 8).

At T1 and T2, participants were asked whether they traveled to other villages. Only a small proportion of forest goers and dwellers (18 to 21%) reported traveling to other villages in the past 30 days (Table 9). This was a bit higher for forest rangers, 35 to 43% of whom reported traveling to other villages within that timeframe, where the area of their primary ranger station was defined as their home “village,” likely due to their work entailing travel throughout the forest. Participants were also asked about whether they had travel companions; almost all individuals traveled with people from the same villages. Travel patterns between timepoints was similar.

### Malaria prevalence

Malaria prevalence was assessed at each timepoint. RDTs were administered to participants who reported having an active fever, which was a total of 43 RDTs throughout the test period, all of which were negative. qPCR was conducted on dried blood spots collected from each participant at all three timepoints. This revealed a number of *P. falciparum* and *P. vivax asymptomatic* infections that are described sequentially below and mapped in supplemental Figs. 1 and 2.

### P. falciparum prevalence

The prevalence of asymptomatic molecularly determined *P. falciparum* infections was similar at approximately 0.5% at both T0 and T1, which dropped to 0.2% in T2 which was an expected result due to declining malaria seasonality throughout the study period (Table 10). Prevalence was higher in Mondulkiri province as compared to Kampong Speu, with similar distribution between males and females. While a prevalence of *P. falciparum* of 4.8% was found in Forest Rangers at T0, no infections were subsequently found in this group. Forest goers and dwellers had roughly the same number of infections across all time points.

When investigating infection locations and travel patterns of infected individuals, *P. falciparum* infections were found to be clustered in villages in both provinces studied. In Mondulkiri province, 67% of cases were concentrated among forest dwellers in Pu Trom and Pu Nhav villages (Table 11). In Kampong Speu, 60% of *P. falciparum* cases were concentrated amongst forest dwellers in two villages as well, Banteay Roka and Banteay Roka Kirisenchey (M). Only three of the nine villages and two of three ranger stations enrolled in Mondulkiri province, and four of nine Kampong Speu villages included in the study had *P. falciparum* infections.

When asked about travel history, none of the individuals with qPCR-positive *P. falciparum* infections reported traveling to other villages within 14 days of positive blood sample collection. In T0 no information was collected about travel to the forest, and this information was only available for some detected cases in T1 and T2, finding that individuals with asymptomatic *P. falciparum* malaria often traveled to the forest. Travel to villages was therefore not a risk factor amongst these cases, while going to the forest was associated with asymptomatic *P. falciparum* infection.

### P. vivax prevalence

The prevalence of asymptomatic *P. vivax* infections detected by qPCR-positive infections was higher than for *P. falciparum*, with an overall prevalence of 4.1% that decreased throughout the study period (122 infections at T0, 78 at T1, and 61 at T2) (Table 12). When comparing between provinces, Kampong Speu had more *P. vivax* infections than Mondulkiri province, males had more infections than females, forest goers and dwellers had roughly the same prevalence of infection across all time points, and no *P. vivax* infections were found in forest rangers.

When investigating locations of *P. vivax* infections, less clustering was observed as compared to *P. falciparum* cases. In Mondulkiri province, Pu Trom village, which had 42% of all *P. falciparum* cases in that province, accounted for 23% of all *P. vivax* cases. A large proportion of *P. vivax* cases were also identified in Andong Kraloeng (28%) and Pu Char (21%), with cases detected in all nine Mondulkiri villages included in the study. In Kampong Speu, the highest proportions of cases were found in Rumduol Thmei (27%) and Peam Lvea (21%) villages, with cases detected in all nine Kampong Speu villages included in the study.

## Discussion

This large-scale serial cross-sectional study identified risk factors for malaria amongst forest-exposed populations in Mondulkiri Province and Kampong Speu Province, finding that in transmission foci in these provinces, participants often worked outdoors as farmers, day laborers, and forest collectors, some (28%) of whom also lived in open structures with two to three walls and a ceiling in their primary residence. Some participants (39%) also reported having a secondary structure they lived in, which was often open, 44% of which had only ceilings. The most common malaria prevention tools used were bednets, wearing long sleeves, and burning insecticide-treated coils. All infections detected during the study were asymptomatic, with clustering in villages observed for *P. falciparum* especially amongst forest dwellers, and no association between cases and self-reported travel to other villages. For *P. vivax* incidence was higher (4% as compared to < 1% for *P. falciparum*), and infections were found in all enrolled villages among forest goers and dwellers, the latter who reported going to the forest more often than other risk groups, with 88% reporting spending time in the forest daily in follow-up surveys. For forest rangers, only two *P. falciparum* cases were detected from one ranger station at the baseline survey, and no *P. vivax* cases were detected.

These findings can be applied directly to malaria elimination efforts in Cambodia. For *P. falciparum*, forest dwellers in villages where infections are found can be targeted for malaria interventions, including Pu Trom and Pu Nhav villages in Mondulkiri and Banteay Roka and Banteay Roka Kirisenchey (M) in Kampong Speu province. *P. falciparum* elimination will require that asymptomatic infections be addressed, a topic that will be discussed in a separate study that infers its prevalence through comparison with data from Cambodia’s malaria information system (MIS). *P. vivax* malaria was found in all study villages, revealing it is still a risk for forest goers and dwellers living in transmission foci. These findings suggest that in these locations, the prevention and treatment of infections can be targeted geographically, a result consistent with occupational and spatial clustering found in another study in Cambodia [12]. Geographical movement however did not present as a risk factor in this study, suggesting that travel between villages is not a major contributor of asymptomatic malaria transmission. In addition to the need to target forest dwellers and goers, this study also found that forest rangers, despite high amounts of time spent in the forest, had much lower malaria prevalence including no *P. vivax* infections found. This could be due to occupational health protective measures provided to rangers as shown by higher reported levels of protection from mosquito bites (80%) compared to other risk groups. Malaria elimination efforts in Cambodia can therefore target forest goers and dwellers in foci at the village level.

When compared to other studies, these findings provide context to efforts to eliminate forest malaria in a variety of settings. An earlier study conducted in 17 villages in Mondulkiri Province from 2017–2018 showed higher levels of PCR-detectable infections, with an incidence of 6.4% for *P. vivax* and 3.0% for *P. falciparum*, approximately two to three times greater than that found in this study [12]. The earlier study also detected hotspots of infection in villages, finding that forest work was associated with malaria. These findings suggest that malaria incidence decreased in the study area since 2017, furthermore confirming that clusters of infection at a village level are a risk factor for malaria in these geographies. This study also provides further insights to those found in a pilot study earlier [14], showing that the use of bednets, wearing long sleeves, and insecticide-treated coils were the most common malaria prevention methods used, and that gaps in protection mostly take place during the day and outside at night.

Targeting these high-risk populations of malaria can combine two approaches. The first is vector control; an evaluation of the distribution and use of forest packs including a topical repellent, a spatial repellent, and insecticide-treated clothes from the parent study is forthcoming and can further inform the selection of vector control tools that can be useful for these populations to prevent malaria. Hopefully forest pack components can overcome the limitations of wearing long sleeves, which was commonly reported in our study, especially when not sleeping indoors, as other Cambodian populations have reported that mosquitoes can bite through clothing [19]. A second approach is chemoprevention, where medicine can be distributed to forest-frequenting populations as intermittent preventive treatment or targeted drug administration to geographic hotspots [20]. This has shown to be effective for forest-going populations in Cambodia when targeting *P. falciparum* malaria [21], although clearing the dormant stages of *P. vivax* is expected to be more challenging [22].

This study had several limitations. It did not specifically include several risk profiles studied in Cambodia, such as illegal loggers [11] and mobile populations that create temporary forest encampments [19]. These risk profiles were studied several years ago however, and malaria endemicity in Cambodia has since decreased substantially, affirming our approach of intervening where malaria hotspots are identified from recently diagnosed cases. Detailed demographic data was not available for the 824 participants enrolled at T1 as these individuals were enrolled to meet sample size requirements. Although those enrolled at T1 represented approximately 30% of the study population, detailed demographic data on 2,111 participants was collected at T0, which we believe is sufficient to represent these at-risk populations in the village foci selected for inclusion.

To support Cambodia’s goals to eliminate malaria, we recommend the immediate application of our findings to local malaria elimination strategies. Forest goers and dwellers should be targeted for prevention and treatment in hotspots where infections are detected. The vector control tools available to these high-risk populations can be expanded, with forthcoming reports on the parent study expected to inform the benefits and challenges on the delivery and uptake of topical repellents, spatial repellents, and insecticide-treated clothing in these locations.

## Supplementary Material

Supplement 1

## Figures and Tables

**Table 1:** Overview of survey data collected at study timepoints

Survey Level		Survey timepoint			Total
		T0	T1	T2	
Village		16	2*	0	18
Household		990	313*	0	1,303
Individual	**Total**	2,111	2,192	2,047	6,350
	**New enrolment**	2,111	824	0	2,935
Survey conducted		Baseline survey	Follow-up survey	Follow-up survey	-

* New villages and households enrolled at T1

**Table 2:** Village summary data

Village characteristic	Location	
	Mondulkiri	Kampong Speu
Villages enrolled	9	9
Number of households per village (average)	199	107
Village population (average)	875	374
Villages accessible by car during the rainy season? (%)	56%	100%
Villages accessible by motorcycle during the rainy season? (%)	100%	100%
Number of Khrom associated with village	2.8	3.2

**Table 3:** Participant demographics

Characteristic			n (%)	Risk group (%)		
				Forest Goer	Forest Dweller	Forest Ranger
*Basic demographic data collected on all new participants (T0 and T1)*						
Total individuals		n (%)	2,935 (100%)	1,093 (37%)	1,787 (61%)	55 (2%)
Province		Mondulkiri	1,510 (51%)	59	45	100
		Kampong Speu	1,425 (49%)	41	55	0
Age		<18	517 (18%)	11	22	0
		18–25	540 (18%)	17	20	2
		26–45	1,253 (43%)	49	37	85
		46–64	563 (19%)	21	18	13
		>65	62 (2%)	2	3	0
Gender		Male	1,434 (49%)	59	41	96
		Female	1,493 (51%)	41	59	4
		Other / not specified	4 (< 1%)	< 1	0	0
*Detailed demographic data collected from new participants at TO only*						
Total individuals		n (%)	2,111 (100%)	730 (35%)	1,339 (63%)	42 (2%)
Ethnic group		Khmer	1,444 (68%)	70	67	81
		Bunong	650 (31%)	28	33	17
		Other	17 (1%)	2	< 1	2
Languages	**Khmer**	Understand spoken	2,100 (99%)	99	99	98
		Speak fluently	2,014 (95%)	95	95	100
		Reading	1,259 (60%)	55	61	100
		Writing	1,210 (57%)	53	58	100
	**Bunong**	Understand spoken	753 (36%)	33	37	43
		Speak fluently	673 (32%)	30	33	38
		Reading	139 (7%)	4	8	7
		Writing	107 (5%)	3	6	2
Household position		Head of household	879 (42%)	54	34	91
		Spouse of head (husband/wife)	434 (35%)	17	23	0
		Child of head (son/daughter)	668 (54%)	24	36	5
		Parent of head (father/mother)	26 (2%)	1	2	0
		Other	104 (9%)	5	5	5

**Table 4:** Participant sources of income (T0)

Income Sources*	Total (%)	Risk group (%)		
		Forest Goer	Forest Dweller	Forest Ranger
Total individuals	2,111 (100%)	730 (35%)	1,339 (63%)	42 (2%)
Farmer	1,687 (47%)	85	79	26
Day labourer	644 (18%)	36	28	0
Forest collector / forager	578 (16%)	37	23	0
Logging	180 (5%)	17	4	0
Market Trader	149 (4%)	7	7	14
Unemployed	59 (2%)	< 1	4	0
Driver / motorbike Taxi	11 (< 1%)	0	1	0
Retired	9 (< 1%)	< 1	< 1	2
Handicrafts (basket weaving, etc.)	3 (< 1%)	< 1	0	0
Other	200 (6%)	4	12	19

* Individuals may list more than one source of income

**Table 5:** Living structure characteristics (T0)

Structure characteristics	Total	Risk group (%)		
		Forest Goer	Forest Dweller	Forest Ranger
**Total individuals n (%)**	2,111 (100%)	730 (35%)	1,339 (63%)	42 (2%)
**Primary living structure**				
**Enclosed room with walls and a ceiling or roof**	71%	74	70	64
**Ceiling and 2–3 walls**	28%	25	30	36
**Only ceiling**	< 1%	1	0	0
**Completely open**	< 1%	< 1	0	0
**Secondary living structure**				
**Have secondary structure in forest or farm?**	39%	52	31	95
**Enclosed room with walls and a ceiling or roof**	33%	25	44	3
**Ceiling and 2–3 walls**	5%	7	3	0
**Only ceiling**	44%	50	39	50
**Completely open**	17%	17	14	48

**Table 6:** Time spent in the forest (T1 and T2)

Time spent in the forest in past week	Total (%) (n = 4,239)	Risk group (%)		
		Forest Goer (n = 1,522)	Forest Dweller (n = 2,622)	Forest Ranger (n = 95)
Did not go to forest	632 (15%)	29%	7%	2%
Went to forest every day	2,889 (68%)	34%	88%	59%
Went to forest but not every day	716 (17%)	37%	5%	39%
Average number of days*	5.1	5.2	4.4	6.4

* For those who went to the forest

**Table 7:** Bednets and hammocks owned by households

Tool ownership		Total (n = 1,303)	Risk group	
			Forest Goer (n = 711)	Forest Dweller (n = 592)
Bednet	Yes	97%	96.5%	97.5%
	How many	2.5	2.4 (range 0–10)	2.6 (range 1–10)
	Treated*	79%	74.8%	83.9%
Hammock net	Yes	66%	64.3%	68.4%
	How many	1.5	1.5 (range 1–5)	1.6 (range 0–9)
	Treated*	84%	81%	89%

* Refers to treatment with insecticides (self reported)

**Table 8:** Travel patterns for purchasing necessities (T0)

Distance travelled	Total (n = 2,111)	Risk group (%)		
		Forest goers (n = 730)	Forest dwellers (n = 1,339)	Forest rangers (n = 42)
Under 500 m	1,686 (80%)	79	84	19
500 m to 2 km	166 (8%)	11	5	33
2 to 5 km	75 (3%)	3	4	2
More than 5 km	184 (9%)	8	7	46

**Table 9:** Travel to other villages during T1 and T2

		Total (%)		Risk group					
				Forest goer		Forest dweller		Forest ranger	
Timepoint		T1	T2	T1	T2	T1	T2	T1	T2
Total Individuals		**2,192**	**2,047**	**801**	**721**	**1,345**	**1,277**	**46**	**49**
Travel to another village in the past 30 days	400 (18%)	406 (20%)	143 (18%)	155 (21%)	237 (18%)	234 (18%)	20 (43%)	17 (35%)
Travel companions	**People from the same village**	1,858 (85%)	1,654 (81%)	610 (76%)	447 (62%)	1,228 (91%)	1,161 (91%)	20 (43%)	46 (94%)
	**People from other villages**	11 (<1%)	5 (<1%)	4 (<1%)	4 (<1%)	2 (<1%)	1 (<1%)	5 (11%)	0 (0%)
	**People from the same and other villages**	64 (3%)	15 (<1%)	12 (1%)	5 (<1%)	31 (2%)	9 (<1%)	21 (46%)	1 (2%)

**Table 10:** Plasmodium falciparum qPCR infections and their distribution

Timepoint		Total	Province		Gender*		Risk group		
			Mondulkiri	Kampong Speu	Males	Females	Forest Goer	Forest Dweller	Forest Ranger
T0	n	2111	1104	1007	999	1108	730	1339	42
	Pos (%)	11 (0.52%)	8 (0.72%)	3 (0.30%)	6 (0.60%)	5 (0.45%)	5 (0.68%)	4 (0.30%)	2 (4.76%)
T1	n	2192	1113	1079	1020	1090	801	1345	46
	Pos (%)	10 (0.46%)	9 (0.81%)	1 (0.09%)	6 (0.59%)	4 (0.37%)	4 (0.50%)	6 (0.45%)	0 (0%)
T2	n	2047	1089	958	935	1026	721	1277	49
	Pos (%)	3 (0.15%)	2 (0.18%)	1 (0.10%)	0 (0%)	3 (0.29%)	1 (0.14%)	2 (0.16%)	0 (0%)

* T0: 4 individuals with unknown gender; T1: 82 individuals with unknown gender; T2: 87 individuals with unknown gender. 0 positives of unknown gender at all three timepoints.

**Table 11:** Residence locations of P. falciparum positive cases

Province	Residency location (village)	Target Group
*Infections detected at T0*		
Mondulkiri	Tu Trom	Forest dweller
	Tu Trom	Forest dweller
	D.A.	Forest goer
	D.A.	Forest goer
	D.A.	Forest goer
	Pu Nhav	Forest goer
	Ranger Station 1	Forest ranger
	Ranger Station 2	Forest ranger
Kampong Speu	Banteay Roka Kirisenchey (M)	Forest dweller
	Banteay Roka Kirisenchey (M)	Forest dweller
	Banteay Roka Kirisenchey (M)	Forest goer
*Infections detected at T1*		
Mondulkiri	Pu Khav	Forest goer
	Pu Khav	Forest goer
	Pu Khav	Forest goer
	Pu Khav	Forest goer
	Tu Trom	Forest dweller
	Tu Trom	Forest dweller
	Tu Trom	Forest dweller
	Tu Trom	Forest dweller
	Tu Trom	Forest dweller
Kampong Speu	Banteay Roka Kirisenchey (M)	Forest dweller
*Infections detected at T2*		
Mondulkiri	Tu Trom	Forest dweller
	Pu Nhav	Forest goer
Kampong Speu	Doung Kraong Meanchey (M)	Forest dweller

**Table 12:** Plasmodium vivax qPCR positive cases and their distribution

Timepoint		Total	Province		Gender*		Risk group		
			Mondulkiri	Kampong Speu	Males	Females	Forest Goer	Forest Dweller	Forest Ranger
T0	n	2111	1104	1007	999	1108	730	1339	42
	Pos (%)	122 (5.8%)	54 (4.9%)	68 (6.7%)	71 (7.1%)	51 (4.6%)	38 (5.2%)	84 (6.3%)	0 (0%)
T1	n	2192	1113	1079	1020	1090	801	1345	46
	Pos (%)	78 (3.6%)	22 (2.0%)	56 (5.2%)	49 (4.8%)	27 (2.5%)	34 (4.2%)	44 (3.3%)	0 (0%)
T2	n	2047	1089	958	935	1026	721	1277	49
	Pos (%)	61 (3.0%)	18 (1.7%)	43 (4.5%)	35 (3.7%)	23 (2.2%)	23 (3.2%)	38 (3.0%)	0 (0%)

T0: 4 individuals and 9 positives with unknown gender; T1: 82 individuals and 2 positives with unknown gender; T2: 87 individuals and 3 positives with unknown gender.

## Data Availability

Data is provided within the manuscript or supplementary information files.
