# Supplementary material for: Malaria risk factors amongst forest going populations in Mondulkiri Province and Kampong Speu Province, Cambodia: a large cross-sectional survey
Source: Res Sq. 2024 Nov 1:rs.3.rs-5291817. Preprint. [Version 1] doi: 10.21203/rs.3.rs-5291817/v1 (PMC11581125; doi:10.21203/rs.3.rs-5291817/v1)
Supplement: Supplement 1 [file NIHPPRS5291817V1-supplement-1.pdf]

Supplementary Files

This is a list of supplementary files associated with this preprint. Click to download.

- BITEP4Supplementaltablesandfigures.pdf
- SupplementalInfoS1VillagedemographicsT0andT1.pdf
- SupplementalinfoS2IndividualT0.pdf
- SupplementalInfoS3IndividualT1.pdf
